# Supplementary material for: Regular walking exercise prior to knee osteoarthritis reduces joint pain in an animal model
Source: PLoS One. 2023 Aug 10;18(8):e0289765. doi: 10.1371/journal.pone.0289765 (PMC10414568; doi:10.1371/journal.pone.0289765)
Supplement: Attachment — Submitted filename: Review.docx [file pone.0289765.s002.docx]

The idea of the manuscript is interesting; however, it needs revision on the English language by a native speaker.

Title: “Regular walking exercise prior to knee osteoarthritis reduces joint pain and synovitis through an upregulation of anti-inflammatory cytokines in the joint”. In my opinion you cannot make the statement that the joint pain reduction was due to an upregulation of anti-inflammatory cytokines once you did not measured protein content, only mRNA expression of 3 different genes. The title should be something like “Regular walking exercise prior to knee osteoarthritis induction reduces joint pain”.

I think the manuscript has few references, please include more references in the manuscript.

Introduction:

Lines 48-49: reference

Lines 69-70: reference

Abstract:

Lines 34-36: it needs to be clearer that there was no difference in the pressure pain threshold BEFORE the injections and that it changes AFTER the injections. It was a little bit confuse for me.

Lines 44-46: walking exercise prior to OA alleviate… alleviate what? Pain? Clarify this on the abstract.

Introduction:

Line 48: Use only exercise for all the sentences where appears physical activity and/or exercise.

Line 77: “using a rat monoiodoacetic acid (MIA)-induced”. Why did you use this model? Please reference this model in this sentence.

Why did you choose to start the exercise program before the monoiodoacetic acid injections and not after?

Why did you stop the exercise program after injections?

In my opinion, there is no two experiments. It is only the continuation of the same experiment. Therefore, do not divide them in two neither in the Methods nor in the Results.

Methods:

Please specify the animal number for each analysis.

Experiment 1:

Lines 99-100 “for the examination of the effect of continuous walking exercise on pain after the onset of knee OA”. It seems you will do the exercise after que onset of knee OA, however, you perform the exercise prior to the injections. It makes the experiment confuse, please reformulate to clarify this matter.

Experiment 2:

Lines 112-113: “We next examined the changes that occur within the knee joint due to loading regular walking exercises.” You actually examined the changes in the knee joint after OA induction with prior exercise or not. Please reformulate this sentence.

You always should, throughout the manuscript, clarify that the exercise was prior to the OA induction. For example in line 115: “regular walking exercise for 6 weeks” you should write “prior regular walking exercise for 6 weeks”. Because it can be confusion to the reader.

Walking exercise with treadmill:

An observation, like humans, animals have a variable maximum oxygen uptake. Therefore, to make future studies more refined, you should perform an Incremental Load Test to every single rat and then you could say all of them walk at the same relative intensity. Because 10m/min could be 40% of the VO2max for one but 30% of the VO2max for another. Once you have the VO2max for every single rat, you separate the ones with similar VO2max and put them to run together.

Line 141: “Rats in the group that in which OA was not induced”. Please write the group name in all this cases (sham group).

Assessment of physical activity:

Lines 156-158: “the measurements were obtained between 15:00 and 18:00, when the light-dark cycle in the rearing room was close to that of the light period and the rats' active period.” Please reference the statement that this is the rats’ active period. I think this result is irrelevant for this manuscript, once there is no difference between groups. Also, you cannot say based only in 1h tracking if the rat was more active or not due to the exercise. Therefore, this result should not be presented in the manuscript.

Statistics:

Why did you used One-way Anova to the other between-group parameters and not two-way anova?

Results:

The results of PPT in figure 2 and figure 6 are the same. Please write all this results together and in 1 figure only.

Merge figure 3 and 7 and write their results in the same topic.

Merge figure 4 and 8 and write their results in the same topic.

Merge figure 5 and 9 and write their results in the same topic.

Line 399: “than the OA group” it is sed OA? Please correct

Discussion:

Line 418: “it suppressed”. It was alleviated, not supressed.

Lines 426-428: please reference the paragraph

Line 434: Please clarify that PRIOR walking exercise did not alleviate, and highlight that you do not know if rats continue to exercise it could be different. This a limitation of the study.

Why you did not verify anti-inflammatory markers through Western blotting? It would reinforces your PCR results. Even though you verified increased mRNA expression of anti-inflammatory markers, you do not know if it was sufficient to increase protein content. If possible, please do the Western blotting analysis from the same mRNA genes you verified.

Lines 473-475: please reference the paragraph

Lines 486-487: “As mentioned above, the expression levels of IL-4 and IL-10 mRNA were increased by 6 weeks of the regular walking exercise”. It is repetitive, please delete.

Line 490: “the expression level of IL-1 mRNA”. Please correct to IL-1beta.

Line 496: “was suppressed by the actions of IL-4 and IL-10”. You cannot infer this, once you did not verify protein content. In addition, you could only say it could be an explanation for these findings.

Lines 506-507: “Second, we were not able to examine the numbers of macrophages after the walking exercise”. Why you were not able?

Lines 517-518: “such as IL-6, tumor necrosis factor alpha (TNF-α), and nerve growth factor in the synovium”. Why you did not verified this genes and proteins? Please verify if it is possible.

Provide foundation sources

Provide authors contributions
