# Supplementary material for: Regular walking exercise prior to knee osteoarthritis reduces joint pain in an animal model
Source: PLoS One. 2023 Aug 10;18(8):e0289765. doi: 10.1371/journal.pone.0289765 (PMC10414568; doi:10.1371/journal.pone.0289765)
Supplement: Attachment — Submitted filename: Response letter to Editor and Reviewers 2 from Authors.docx [file pone.0289765.s004.docx]

Tanja Grubić Kezele, Ph.D., M.D.

Academic Editor

We greatly appreciate you and reviewers’ specific comments and opportunity to resubmit with appropriate corrections. We revised our manuscript as reviewer2 pointed out. We have sorted out the descriptions of the considerations that were redundant.

Each of your comments have been answered below.

**Reviewer #2:**

We greatly appreciate your comments that clarified the problems of the manuscript, and we are thankful for your thoughtfulness and kindness. We revised our second manuscript as you pointed out. Each of your comments have been answered below.

1. **Walking exercise with treadmill:** **In the next studies you could perform a incremental load test until exhaustion, it will give to you the max aerobic power based on the maximum velocity reached on the test. Than you can use a percentage from the maximum velocity and submit the rats to run in the treadmill based on individual test. You do not need more equipment, just a treadmill.**

Thank you for your advice. Based on your advice, we revised the manuscript on the study limitations.

2. **Results:**

**You did not mention on the Results the differences in the PPT between Sed + OA and Ex + OA, however it was one of the main conclusions of the experiment. Please make it clear in the results description.**

We apologize that we have not been able to make enough revisions to the results. We　have added the results the differences in the Sed + OA and Ex + OA groups in line with your points.

3. **Discussion:**

**The discussion is well written. However, it is too extensive. Please, be more direct to the point, shortening the discussion.**

Thank you for your advice. Based on your advice, we revised the manuscript on the discussion.
